# Dialysis Modality Education Timing and Home Dialysis Uptake: A Quality Improvement Study

**DOI:** 10.1016/j.xkme.2024.100898

**Published:** 2024-09-05

**Authors:** Declan (Shiyu) Lu, Mishal Akhtar, Lisa Dubrofsky, Bourne L. Auguste

**Affiliations:** 1 Division of Nephrology, University of Toronto, Toronto, Ontario; 2 Department of Medicine, Womens’ College Hospital, Toronto, Ontario; 3 Department of Medicine, Sunnybrook Health Sciences Centre, Toronto, Ontario; 4 Centre for Quality Improvement and Patient Safety, University of Toronto, Toronto, Ontario

**Keywords:** Home dialysis, peritoneal dialysis, home hemodialysis, education, quality improvement

## Abstract

**Rationale & Objective:**

Investigating the effect of a quality improvement intervention aimed at enhancing the choice of home dialysis among patients through improved educational sessions on dialysis modalities.

**Study Design:**

A new referral protocol initiated on September 15, 2022, sought to direct patients with advanced kidney disease to modality education sessions. This protocol involved an updated referral form and process, requiring nephrologists to refer patients with an estimated glomerular filtration rate below 15 mL/min/1.73 m^2^ or specified Kidney Failure Risk Equation scores to modality educators for education. The impact was measured by the uptake of the education and the choice of home dialysis by patients.

**Setting & Participants:**

The study took place at Sunnybrook Health Sciences Centre in Toronto, Canada, involving 532 patients across 1,723 clinical encounters from October 2019 to June 2023.

**Predictor:**

The intervention was predicted to lead to an increase in both the number of patients receiving modality education and those choosing home dialysis.

**Outcomes:**

The primary outcome measured was the selection of home dialysis following modality education, with a secondary focus on the proportion of patients educated post intervention.

**Analytical Approach:**

Statistical process charts were used for monitoring changes in education uptake and home dialysis selection rates following the intervention.

**Results:**

After implementing the standardized referral system, the proportion of patients receiving modality education increased from 27.1%-56.7%. However, the rate of selecting home dialysis remained constant at 50.9%. Overall home dialysis prevalence at our center averaged 19.6%, remaining lower than the provincial average of 24.4% by the end of the study period.

**Limitations:**

The study was limited to 1 center, without evaluating patient satisfaction or dissecting the complexity of educational content and delivery.

**Conclusions:**

We succeeded in boosting education rates but failed to achieve higher home dialysis choice rates, possibly owing to the complexity involved in modality choices. We plan to further investigate the factors influencing patient choices during modality education to better promote home dialysis selection.

## Plain-Language Summary

The study focused on whether teaching patients more about their dialysis options would encourage them to choose home dialysis. A new system was introduced at an academic hospital in Toronto, requiring doctors to refer patients with advanced kidney disease to educational sessions. The aim was to see if patients who learned more about dialysis would be more likely to manage their treatment at home. The result was more patients received these educational sessions, but this did not lead to more of them choosing home dialysis. Future research must investigate what other factors influence patients’ decisions to consider dialysis treatments at home.

## Introduction

It is expected by 2030 that the demand for dialysis will significantly increase, with an estimated 15.4 million people globally requiring treatment.^1^ Home dialysis, encompassing modalities such as peritoneal dialysis (PD) and home hemodialysis, stands out as a key strategy in addressing this need. This alternative to in-center hemodialysis offers a more favorable hemodynamic profile with gentler fluid removal, with clinical benefits including improved blood pressure control with home hemodialysis and preservation of residual kidney function with PD.^2^, ^3^, ^4^ In addition, for health care systems grappling with financial constraints, home dialysis represents a cost-effective alternative, yielding significant savings compared to in-center hemodialysis through reduced clinical infrastructure and operational costs.^5^, ^6^, ^7^, ^8^, ^9^

Despite the compelling case for home dialysis, numerous barriers at various levels impede more wide-scale adoption. Recognizing these challenges, the KDIGO 2018 Controversies Conference underscored the essential role of enhanced education on dialysis modalities for both patients and caregivers.^10^ Evidence from systematic reviews suggests an association between early predialysis education and improved patient outcomes, particularly highlighting that delays in education are linked to higher mortality rates in the first year of dialysis.^11^ Furthermore, patients who receive early education are more likely to select home dialysis as their treatment modality.^12^ Individualized educational programs are essential, aiming to address the diverse needs across patient populations, thereby ensuring that more individuals can benefit from the improved autonomy and quality of life afforded by home dialysis.^13^, ^14^, ^15^

Ontario, the province with Canada’s highest population, is promoting increased home dialysis uptake because it confronts escalating needs for kidney care services, with over 12,000 patients currently undergoing dialysis. The Ontario Renal Network, a provincial regulatory body that oversees kidney care under the auspices of the provincial government, has established a forward-looking goal to have at least 25% of patients with end-stage kidney disease receive home dialysis.^16^^,^^17^ This approach is not only a response to the escalating need but also a recognition of the intrinsic benefits home dialysis brings to patient care and the health care system at large.

The Ontario Renal Network supports predialysis education, particularly when patients near a crucial 2-year risk threshold for advancing into end-stage kidney disease, as assessed by the Kidney Failure Risk Equation (KFRE). This regulatory body advocates for the provision of detailed modality education to patients in predialysis clinics with a 2-year KFRE value of 20%-40%.^17^ Despite this recommendation, a review of the practices within our Multi-Care Kidney Clinic (MCKC) at Sunnybrook Health Sciences Centre in Toronto, Ontario, from January 1, 2018, through September 30, 2019, revealed that a strikingly low percentage (27.1%) of patients with a 2-year KFRE value of ≥40% had been recipients of such education. In addition, a Provincial Site Visit Report highlighted that early modality education with recurring conversations has mobilized patients to adopt home dialysis.^16^ This method promotes timely and appropriate education and preparation while patients progress toward kidney replacement therapy.^17^^,^^18^

An Ontario Renal Network Provincial Site Visit Report revealed that timely education on treatment options encourages patients to choose home dialysis.^16^ Observing this report, we considered a potential link between suboptimal education rates and low home dialysis adoption rates. Our hypothesis was that low home dialysis adoption rates could be due to gaps in the educational offerings within our program. We proposed that by improving the delivery of our existing education, without altering its content, we could address the shortcomings pointed out by our provincial oversight agency, potentially leading to more patients choosing home dialysis. Consequently, we initiated a quality improvement (QI) initiative to improve modality educational rates among our patients, aiming to address this identified gap within our program.

As a result, the primary aim was to increase the proportion of prevalent educated patients with a KFRE value of ≥40% by 75% from baseline (a goal of at least 60% prevalent patients to get educated) by June 30, 2023. The secondary aim was to increase the home dialysis adoption rate of prevalent patients who received education in the clinic by 75% (a goal of at least 50% of educating patients to choose home dialysis) by June 30, 2023. The project was led by a nephrologist with QI and home dialysis training with the support of program leadership given the low rates of home dialysis choice and adoption rates. This project aligned with organizational priorities to address educational and home dialysis gaps within the program.

## Methods

### Ethical Issues

This QI project was registered at Sunnybrook Health Sciences Centre in Toronto, Canada (Sunnybrook Health Sciences Centre QI-161). Research ethics review was not required because the project met criteria for exemption from such a review based on our institutional process for confirming that the project was deemed improvement in quality and not human subject research. This investigation was conducted, evaluated, and reported in concordance with the methodological standards prescribed by the Standards for Quality Improvement Reporting Excellence guidelines.^19^

### Setting

Patients are seen within the MCKC once at least every 3 months and more frequently as kidney disease progresses. The clinics at Sunnybrook Health Sciences Centre include a nephrologist, pharmacist, dietitian, social worker, and nurses who serve as dedicated modality educators. Our modality education approach involves the use of educational slides and one-on-one meetings with patients and caregivers, either in person or virtually, typically presented over a period of 60-90 minutes. It is important to note that the content and duration of the educational sessions remained unchanged throughout the study period. In addition, modality educators providing the education were unchanged throughout the study period.

Our study excluded those who declined any form of kidney replacement therapy for conservative care, as well as patients under active treatment at our glomerulonephritis clinic.

### Planning the Intervention

Given that patients in this clinic were seen at least once every 3 months, we conducted an audit of data on a quarterly basis to track education and choice rates. The chart audit was done using our electronic medical record system (Accuro) to identify data around demographics, KFRE values, estimated glomerular filtration rates, education documentation, and choice rates. The quarters were defined as follows: January 1 to March 31: quarter 1 (Q1); April 1 to June 30: quarter 2 (Q2); July 1 to September 30: quarter 3 (Q3); October 1 to December 31: quarter 4 (Q4).

**Figure 1:**
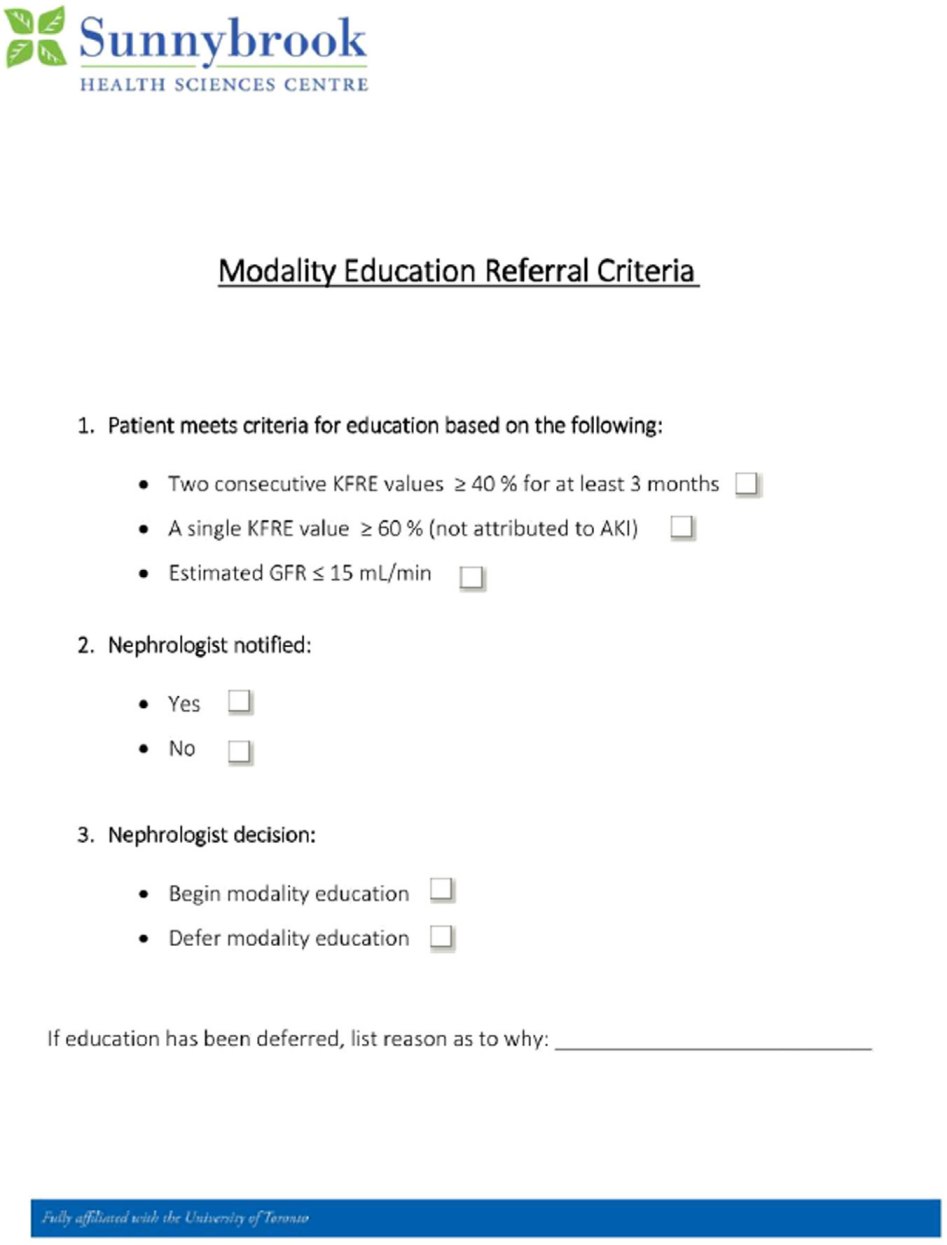
The modality education referral criteria form created by the team. Abbreviations: AKI, acute kidney injury; GFR, glomerular filtration rate; KFRE, Kidney Failure Risk Equation.

To optimize patient education, we engaged in discussions with modality educators and nephrologists, leading to a consensus on an education initiation process. The talks revealed hesitation about relying solely on the KFRE for referrals, as it may not fully capture the nuances of disease in patients with low albuminuria levels. Consequently, we developed an educator-driven referral pathway, establishing a form that included a provision for patients with an estimated glomerular filtration rate under 15 mL/min/1.73 m^2^, even if their KFRE value was below 40%. Furthermore, it was recognized that acute incidents could inflate KFRE percentages rapidly in chronic kidney disease cases. Thus, we adopted a criterion requiring 2 KFRE readings over 40%, spaced 3 months apart, to qualify for education referral. A single KFRE measurement exceeding 60%, not due to acute kidney injury, also qualified for immediate education. Before each MCKC, educators would review patient lists and insert referral forms into the electronic medical records of those who met these criteria. This electronically prompted the treating nephrologist to indicate whether the patient should undergo modality education or not. If the treating nephrologist felt the patient should not undergo education, a reason was required (Fig 1). To study the implementation of our intervention, we collected data across 15 quarters between October 1, 2019, and June 30, 2023. Our intervention was implemented on September 15, 2022.

### Methods of Evaluation

For evaluation, our main process measures centered on the percentage of patients with a KFRE value of at least 40% who received education in the clinic. Our primary outcome measure was the proportion of patients opting for home-based therapy postmodality education. Both process and outcome measures were analyzed using statistical process control charts. In addition, the overall adoption rates of home dialysis were monitored as a secondary, lagging indicator. To assess the effectiveness of our intervention, we randomly audited 10 charts in the first 2 months after implementation to verify the proper use of referral forms, serving as our fidelity measure. Finally, modality educator satisfaction and changes in clinical practice were used as balancing measures, evaluated through an anonymous survey completed by the educators.

### Analysis

In our study, we employed statistical process control charts to monitor and analyze the extent to which targets were met for modality education and home dialysis choices over time. These charts combine chronological analysis with statistical significance tests, enabling us to assess the efficiency and long-term viability of our processes.^20^ To construct these charts, we first calculated the average (control limit) value over the study period. We then determined the upper and lower control limits, set at 3 standard deviations (SDs) above and below this mean, using p-chart formulas based on the binomial distribution.

Our assessment of patient education and home dialysis selection was distributed into quarterly phases. We tested a null hypothesis assuming random variation in education and choice rates, contrasted with an alternative hypothesis attributing significant shifts to our targeted intervention, notably the inclusion of a referral form in patient electronic medical records. In our QI analysis, we examined the data for “special-cause” variation—evidence of nonrandomness in the results. “Special-cause” variation occurs when changes in data signal a specific event or cause, such as a new policy, procedure, or intervention that differs from the expected variability of a process. Indicators include a run of 8 consecutive data points above or below the mean, 6 consecutive points trending up or down, 2 out of 3 points lying beyond 2 SDs from the mean, or a single point more than 3 SDs from the mean—known as a 3 sigma violation. To construct our charts, we used Microsoft Excel–based QI Macros software (2019 KnowWare International Inc), a tool designed for such analytical tasks.

## Results

### Patient Characteristics

During the study period, we assessed 532 unique patients with 1,723 clinical encounters in the MCKC program. The mean age was 68.16 ± 16.81 years, and 60.2% of patients were men (Table 1). The leading cause of chronic kidney disease in the clinic was diabetes in 50% of patients. The mean estimated glomerular filtration rate of patients was 14.46 ± 5.71 (SD) mL/min/1.73 m^2^. Two-hundred thirty-six (44.37%) patients received kidney replacement therapy by the end of the study period. The median time from initial modality education to first kidney replacement therapy was 349 days. Following education on treatment modalities, 34.2% of patients were undecided, whereas 23.7% chose in-center hemodialysis, 22.9% selected PD, 9.2% preferred conservative care, 8.7% opted for a transplant, and 1.3% decided on home hemodialysis.

**Table 1:** Baseline Characteristics

Age (y)	68.16 ± 16.81^a^
Male, % (n)	60.2 (320)
eGFR	14.46 ± 5.71^a^
No. of days from initial education to dialysis start, median (IQR)	349 (103-676.5)
Cause of CKD, n (%)	
Diabetes	266 (50)
Hypertension	74 (13.9)
Glomerulonephritis	67 (12.6)
Renovascular disease	63 (11.7)
Polycystic kidney disease	11 (2.1)
Obstructive uropathy	18 (3.4)
Other	34 (6.4)
Patient Modality Preferences After Education, n (%)	
Undecided/no final decision	182 (34.2)
Home hemodialysis	7 (1.3)
Peritoneal dialysis	122 (22.9)
In-center hemodialysis	126 (23.7)
Transplant	46 (8.7)
Conservative kidney care	49 (9.2)

Abbreviations: CKD, chronic kidney disease; eGFR, estimated glomerular filtration rate; IQR, interquartile range.

a Mean ± standard deviation.

### Pre-intervention

**Figure 2:**
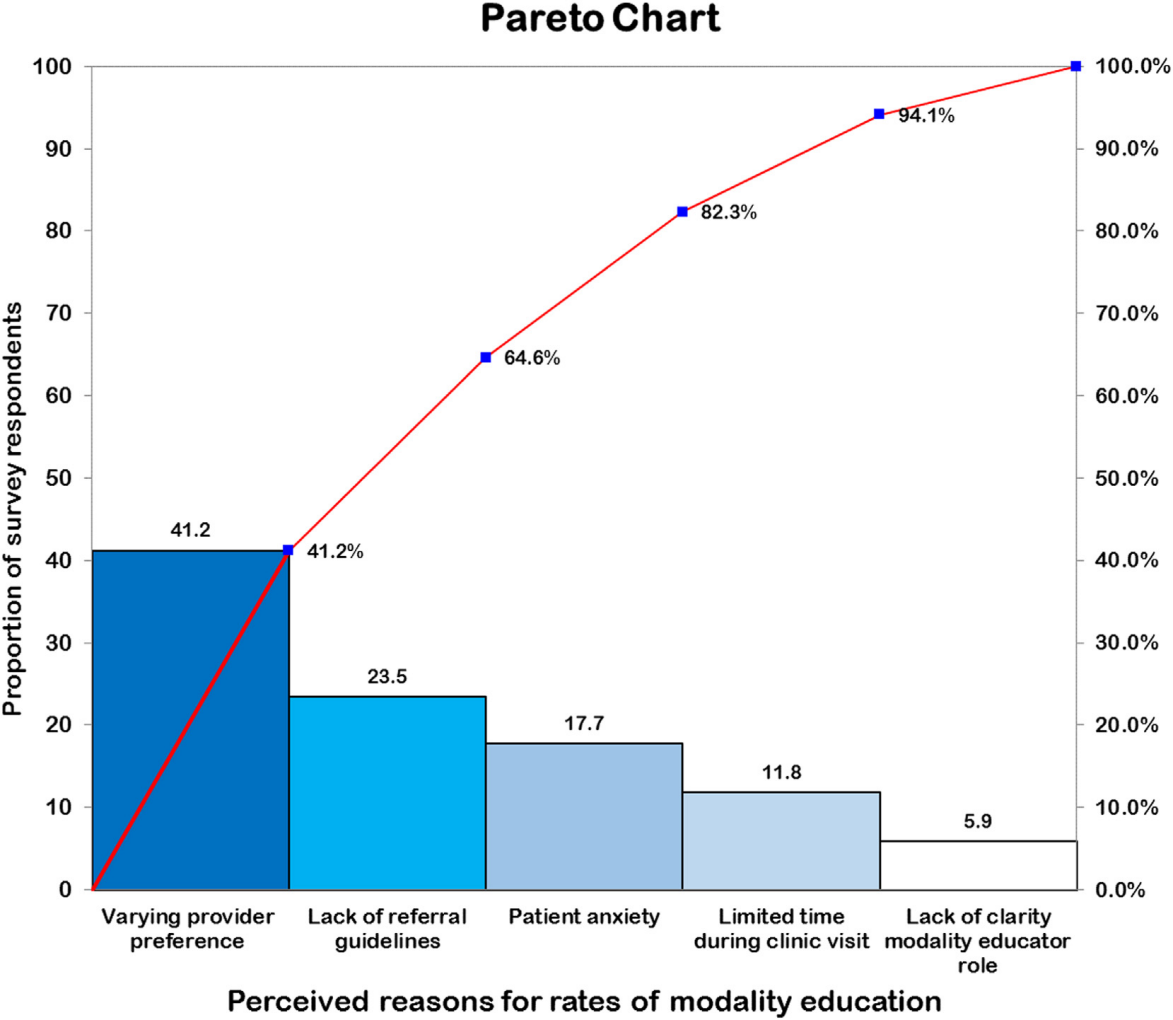
A Pareto chart demonstrating perceptions from various team members accounting for low rates of modality education in clinics.

An initial data audit was conducted across 7 quarters between January 1, 2018, and September 30, 2020, to establish baseline rates of modality education and home dialysis choice before the implementation of any intervention as well as to demonstrate feasibility of the collection process. After completion of the audit, we presented our data to all MCKC team members to demonstrate the existing gap and opportunities for improvement within our program. We demonstrated that our practice fell short of Ontario Renal Network recommendations in terms of the timing of education within our program. Specifically, 27.1% of patient encounters with a KFRE value of ≥40% had received modality education. As a result, to better understand the nature of the problem, we conducted anonymous surveys (Survey Monkey) of all MCKC staff including nephrologists. The results of the survey were used to formulate a Pareto chart (Fig 2) such that we could prioritize our intervention on the most frequently cited concern by survey respondents.

### Outcomes

**Figure 3:**
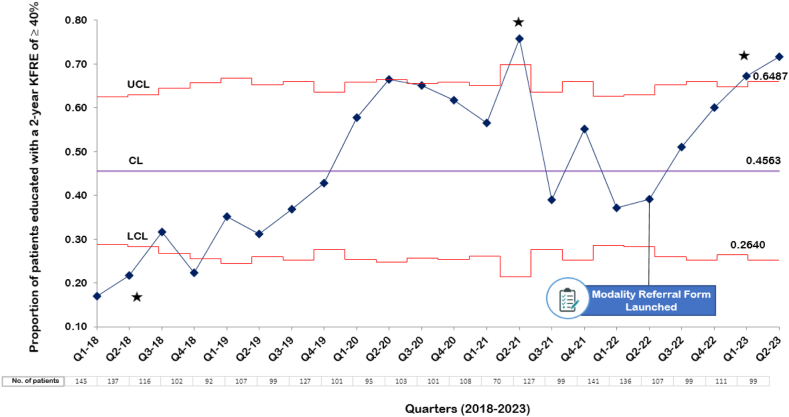
The proportion of patients with a 2-year Kidney Failure Risk Equation (KFRE) of at least 40% received modality education across various quarters. The control limit (CL), upper control limit (UCL), and lower control limit (LCL), are plotted as the mean, 3 standard deviations above and below the mean, respectively. Three instances of special-cause variation are noted in this chart (★). The special-cause variation seen after the modality form was launched demonstrates 2 out of 3 consecutive measurements being 2 standard deviations from the mean.

In the process measures, our target proportion of patients educated in the clinic was achieved and sustained after the intervention was launched. The proportion of patients who received modality education gradually increased from our initial baseline rate of 27.1% to an average of 45.6%. In the 5 quarters of data collected after the implementation of our intervention, the proportion of educated patients was 56.7%. In addition, the change in proportion of educated patients was associated with special-cause variation, as identified in the statistical process control chart (Fig 3). Specifically, 3 instances of special-cause variation were detected. Notably, 2 of these instances occurred after the implementation of our intervention, characterized by at least 2 out of 3 consecutive measurements being more than 2 SDs above the mean. In addition, following the implementation of the referral form in clinical practice, 2 measurements were observed to exceed the upper control limit of 0.6487. From Q3-2019 to Q3-2020, we noticed an increase in the rates of patient education, which may indicate a Hawthorne effect. This uptick coincided with a strategic push in our nephrology program to promote home dialysis, marked by the recruitment of a nephrologist dedicated to increasing its adoption and improving patient education.

**Figure 4:**
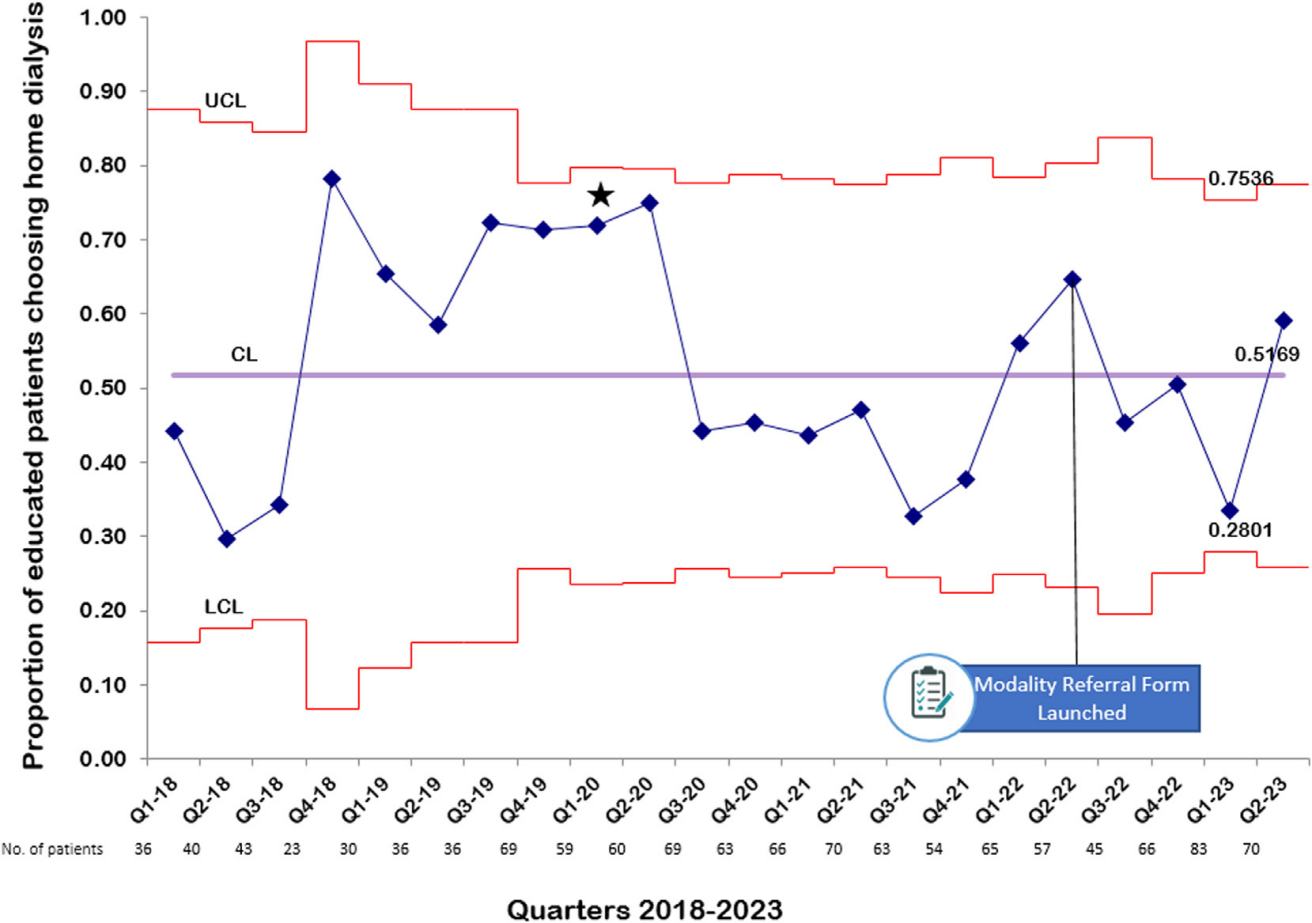
The proportion of educated patients who chose home dialysis as their preferred modality. The control limit (CL), upper control limit (UCL), and lower control limit (LCL) are plotted as the mean, 3 standard deviations above and below the mean, respectively. The star denotes instances of special-cause variation. One special-cause variation (★) with 2 out of 3 consecutive measurements being 2 standard deviations from the mean occurring before the intervention and coincided with the beginning of the coronavirus disease 2019 pandemic.

**Figure 5:**
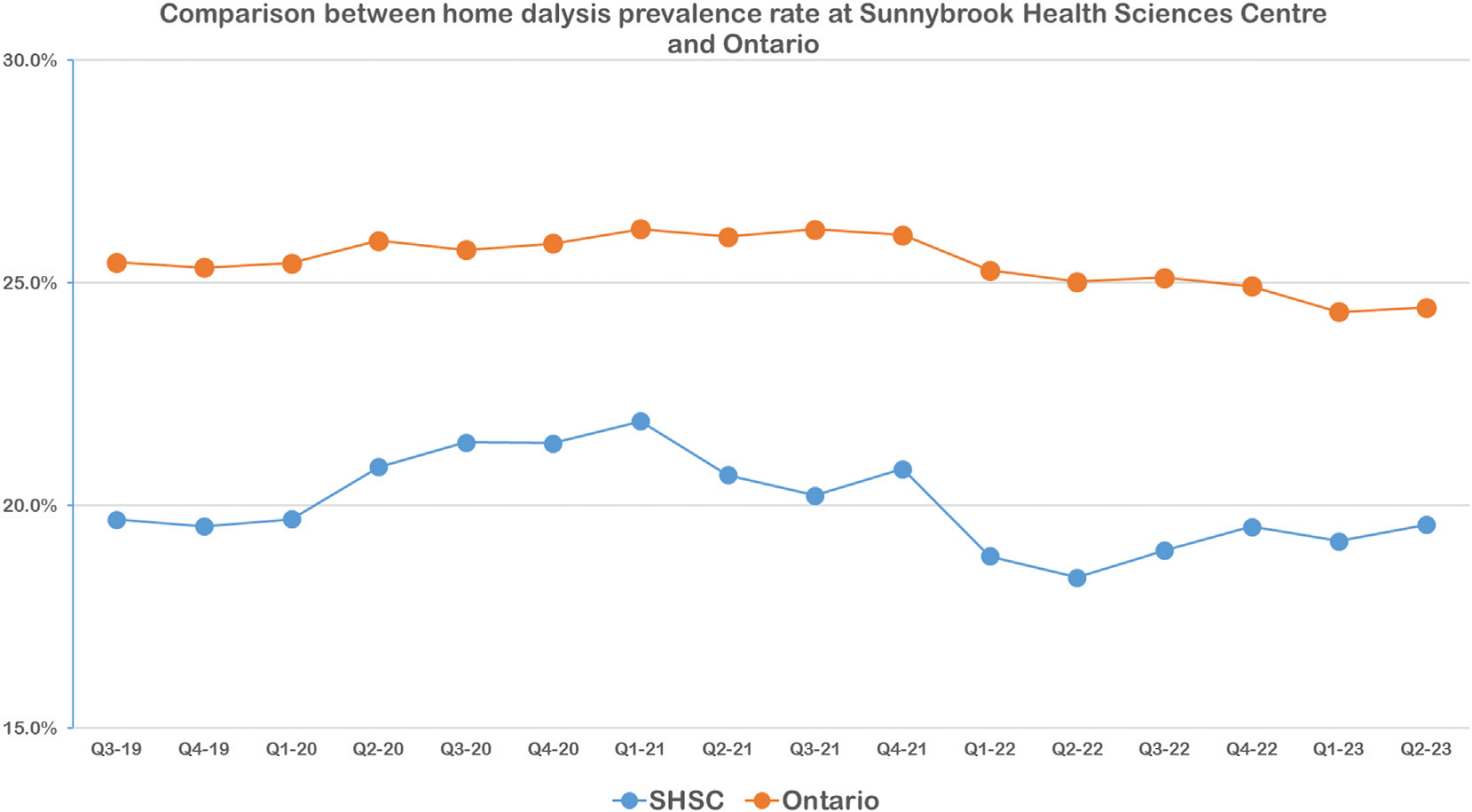
A comparison between home dialysis prevalence rates at Sunnybrook Health Sciences Centre (SHSC) and within the province of Ontario from the third quarter of 2019 to the second quarter of 2023 (data source: Ontario Renal Network insights report).

The statistical process control chart for home dialysis choice rates among educated patients did not demonstrate any special-cause variation before or after the implementation of our modality referral form. The mean choice rate was 51.7% across the study period compared to 50.9% after the implementation of our intervention (Fig 4). There was a special-cause variation detected, but this occurred in 2020, which coincided with the coronavirus disease 2019 pandemic. No special-cause variation was seen after the implementation of our modality referral form. Home dialysis rates within our program and across the province maintained a consistent level, showing no substantial changes, although a slight downward trend became noticeable following Q4-2021 (Fig 5).

## Discussion

Despite successfully increasing predialysis modality education rates, the rate of home dialysis selection and prevalence within our program did not significantly increase compared to baseline before intervention. This suggests that patient decisions regarding dialysis modalities are influenced by a complex interplay of factors, as has been reported in a recent Canadian qualitative descriptive study.^21^ Observational data indicate that the duration of therapeutic relationships with health care providers may play a crucial role in these decisions. The examination of United States Renal Data System data has shown that patients followed by providers for more than 12 months are more likely to choose home dialysis.^12^ In addition, observational data from Europe have also shown that nearly 50% of patients who receive effective predialysis education, support, and training adopted self-care dialysis.^22^ This highlights the importance of building an ongoing therapeutic relationship with patients who may overcome anxiety to pursue home dialysis. Besides the length of the provider–patient relationship, other factors such as individual health profiles, psychological readiness for home care, family and social support systems, financial considerations, and personal beliefs and preferences about health care significantly influence the choice of dialysis modality.^23^, ^24^, ^25^ In addition, the way education is delivered to patients, families, and caregivers is also of critical importance. The 2018 KDIGO controversies conference report on dialysis initiation, modality choice, access, and prescription highlighted the importance of presenting educational material to patients in an unbiased manner.^10^ Observational data among incident home dialysis patients suggest that there are differences in learning styles, which may have an impact on knowledge acquisition and retention among patients.^14^^,^^15^

Our analysis, using a Pareto chart for root-cause assessment (Fig 2), revealed that the main impediments to timely patient education were referral uncertainties and inconsistencies in the initiation of education, echoing findings from a recent US study, which identified variable clinical practices, and sporadic nephrologist engagement in the education process.^23^ We introduced a referral form that reduces reliance on the KFRE by requiring a more robust, dual-checkpoint approach, which is as follows: 2 KFRE readings above 40%, spaced at least 3 months apart, to mitigate the impact of acute kidney injury episodes on chronic kidney disease management. This approach underpins the necessity of clear, consensus-driven guidelines that synchronize clinical assessments with educational outreach, ensuring patients receive relevant information at the optimal time without premature or delayed intervention.

In our study, we observed a potential Hawthorne effect in the data spanning from Q3-2019 to Q3-2020 (Fig 3). Specifically, there was a marked increase in the proportion of patients receiving education on dialysis modalities, increasing from 0.368-0.665. The Hawthorne effect has been well described in QI studies, where the announcement of an initiative may be modulate behavior.^26^ In addition, there was a brief increase in the choice rates for home dialysis, particularly from Q3-2019 to Q4-2019. These changes coincided with the hiring of a new nephrologist who was specifically focused on the growth of home dialysis modalities. The announcement of a QI project aimed at addressing the low education rates within the program likely influenced this shift. These factors combined suggest that the observed changes in patient education and modality choice were, at least in part, a response to the altered dynamics and increased focus within the program. However, it is important to note that this shift was not sustained over time, indicating that the initial response to the hiring of a new nephrologist and announcement of a QI project may have temporarily modulated behavior within the program, but did not lead to long-term changes.

In Fig 4, there was a transient “special-cause” variation in home dialysis choice rates at the onset of the coronavirus disease 2019 pandemic. This was likely driven by recommendations to reduce at-risk individuals and alleviate the strain on hospital and other health care resources at the time.^16^^,^^27^^,^^28^ Despite this, the increase in home dialysis choice rates did not substantially affect the long-term prevalence of home dialysis use. The subsequent modest decline in home dialysis prevalence rates, shown in Fig 5 beyond Q4-2021, possibly corresponds to an increase in kidney transplant activity as services restarted with some easing of early pandemic restrictions.

It should be noted that our program has a relatively high prevalence of patients receiving PD, mirroring provincial rates, suggesting a ceiling effect in which the potential for increase is constrained by near-full patient engagement. A ceiling effect here means our high PD adoption is close to a practical maximum, making substantial growth difficult owing to patient suitability and program limitations. This could also partly explain why higher education rates may not immediately translate into higher prevalence rates for home-based therapies.

The limitations of our study are multifaceted and warrant a thorough examination. First, it was conducted within a single center, raising questions about the broader applicability of our findings to diverse populations across various demographic and geographic regions. This limitation restricts our ability to predict if our results would hold true in differing clinical environments. Second, a notable omission in our methodology was the lack of a robust mechanism to measure patient satisfaction posteducational intervention. Patient satisfaction is a critical determinant in the long-term viability and success of an intervention. Without this measure, we missed the opportunity to learn from patient experiences and feedback, which could be instrumental in refining and optimizing educational strategies. Last, and perhaps most important, our study did not explore the intricacies of how educational content is delivered, nor did we examine the preparedness of patients and their families, which can vary owing to factors such as anxiety, readiness, and social circumstances. These elements might greatly affect decision making and could also account for the observed discrepancy between the enhanced rates of education and the unchanged adoption of home dialysis that we observed. Our educational strategy was not tailored to individual patient needs and lacked assessment of the real-world effectiveness of the content delivered. Table 1 revealed that even after education, over one-third of patients remained undecided on their modality choice, suggesting that enhanced educational efforts do not necessarily lead to decisive patient decision making. Future research should delve into these nuances, seeking to understand what educational strategies are most effective for whom, why, and when. By dissecting these dynamics, we can develop a more nuanced understanding of patient decision-making processes and tailor educational interventions that are not only informative but also align with the personal context and readiness of patients and their support system.

In conclusion, this study shows that the application of QI strategies can effectively enhance modality education rates in predialysis clinic settings. However, it is critical to acknowledge that elevated educational engagement does not directly result in an increased selection of home dialysis options by patients. The decision-making process for home dialysis is influenced by a myriad of factors, necessitating a more nuanced approach to bolster its acceptance. It is imperative that future research delves into the complex interplay between educational content, the timing of its delivery, and patient decision making. A comprehensive understanding of these elements is vital to empower patients, caregivers, and health care practitioners in making informed decisions pertaining to kidney replacement therapies.
